# An integrated system from microscopy to AI for real-time object detection in endometrial cytology

**DOI:** 10.1016/j.jpi.2025.100541

**Published:** 2025-12-31

**Authors:** Mika Terasaki, Shun Tanaka, Ichito Shimokawa, Etsuko Toda, Shoichiro Takakuma, Yusuke Kajimoto, Shinobu Kunugi, Akira Shimizu, Yasuhiro Terasaki

**Affiliations:** a Department of Analytic Human Pathology, Nippon Medical School, 1-25-16, Nezu, Bunkyo-ku, Tokyo 113-0031, Japan; b Faculty of Medicine, Nippon Medical School, 1-1-5, Sendagi, Bunkyo-ku, Tokyo 113-8602, Japan; c Laboratory for Morphological and Biomolecular Imaging, Nippon Medical School, 1-25-16, Nezu, Bunkyo-ku, Tokyo 113-0031, Japan; d Division of Pathology, Nippon Medical School Musashi Kosugi Hospital, 1-383, Kosugimachi, Nakahara-ku, Kawasaki 211-8533, Kanagawa, Japan; e Division of Pathology, Nippon Medical School Hospital, 1-1-5, Sendagi, Bunkyo-ku, Tokyo 113-8602, Japan

**Keywords:** Artificial intelligence, Endometrial cytology, YOLOv5x, Object detection, Real-time support, Pathology workflow integration, Digital pathology

## Abstract

Endometrial cytology, which is minimally invasive and available as an outpatient procedure, is widely used in Japan for early detection of endometrial cancer, but its diagnostic process is time-consuming and requires expert diagnosticians. We developed a real-time artificial intelligence (AI)-assisted system using a standard microscope, a charge-coupled device (CCD) camera, and the You-Only-Look-Once version 5x (YOLOv5x) (a well-established object detection model) to support endometrial cytology screening in resource-limited settings. A total of 146 pre-operative cytology cases were collected, and the model was trained to detect abnormal cell clusters. The system was evaluated in real-time using a CCD camera, and its diagnostic performance was compared with that of three pathologists and four medical students. In an independent test of 20 cases, the AI model achieved an accuracy of 85%, showing promising performance comparable to the average accuracy of 75% among human evaluators. Furthermore, the median diagnostic time was reduced by approximately 45% with AI assistance. The impact of AI support varied by user expertise, with notable improvements among non-specialists. This proof-of-concept study demonstrates the feasibility and potential of affordable, real-time AI support for endometrial cytology using widely available equipment. Further validation with larger, multicenter datasets is warranted to confirm the generalizability and clinical utility of this approach.

## Highlights

• Real-time AI support for endometrial cytology is feasible with standard hardware.
• AI assistance reduced median diagnostic time by approximately 45%.
• AI improved diagnostic accuracy, particularly for non-specialist evaluators.

## Introduction

Endometrial cancer is the most common gynecological malignancy in Japan and the second most prevalent worldwide, with incidence rising due to demographic factors such as aging and obesity.^1^, ^2^, ^3^, ^4^ Early detection is critical for improving prognosis.^5^ Whereas transvaginal ultrasound and biopsy are internationally established, endometrial cytology is widely used in Japan as a minimally invasive, outpatient-friendly screening method with high specificity and positive-predictive value.^6^, ^7^, ^8^, ^9^

However, the digitization of cytological slides remains challenging due to the high cost and complexity of whole-slide imaging (WSI) systems, as well as a shortage of expert diagnosticians.^10^, ^11^, ^12^ Unlike histology and cervical liquid-based cytology slides, many cytology preparations contain cellular aggregates forming complex three-dimensional structures, posing significant autofocusing challenges for WSI scanners.^12^

In recent years, artificial intelligence (AI) technologies, particularly real-time object detection models such as the You Only Look Once (YOLO) family, have become widely established in the medical imaging field.^13^ Whereas these models have achieved success in radiology and other pathology domains,^13^ their application is often predicated on images digitized by expensive WSI systems. Although AI assistance in cytology has been reported for applications such as cervical cytology,^14^ to our knowledge, no prior study has implemented a real-time AI-assisted system for endometrial cytology—which is characterized by complex three-dimensional cell clusters that pose challenges for WSI—using only a standard microscope and an affordable charge-coupled device (CCD) camera.

This study addresses this gap by developing and evaluating a real-time AI-assisted system for endometrial cytology. Whereas endometrial cytology is a practice widely utilized in Japan and some other countries, the technical challenges it presents—such as complex 3D cell clusters—make it an ideal test case for developing a robust, real-time AI framework adaptable to other cytological applications worldwide. Our aim is to provide visual guidance for non-expert users and improve workflow efficiency. By combining the cost-effectiveness of endometrial cytology with affordable AI technology (standard microscope + CCD camera), this approach offers a practical solution for resource-limited settings.

## Materials and methods

### Study design and case selection

A total of 146 pre-operative endometrial cytology cases, collected between April 2017 and March 2023 at a single university hospital in Japan, were included. The final classification of malignant (72 cases) or benign (74 cases) was determined by surgical pathology specimens. The malignant cases included predominantly endometrioid carcinoma (grades 1–3) and serous carcinoma, whereas benign cases were leiomyomas. Detailed case distributions are summarized in Supplementary Table S1.

**Fig. 1:**
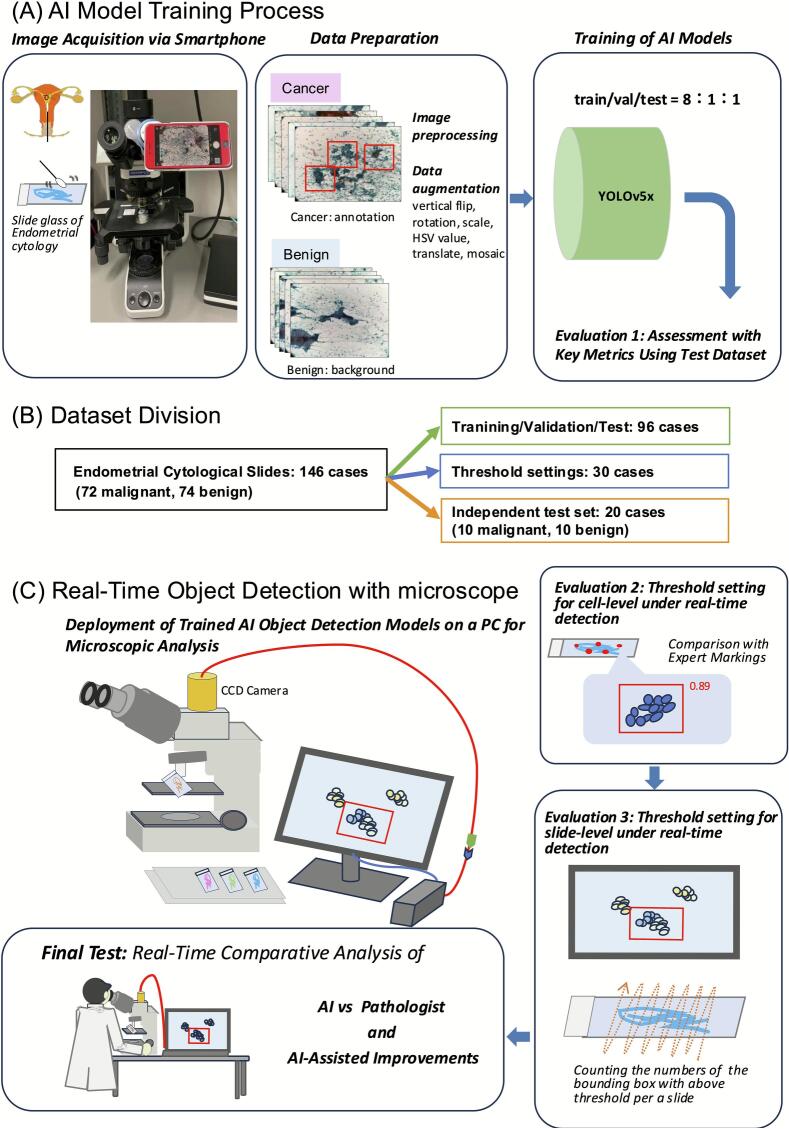
Study overview. The analysis of endometrial cytology slides using You-Only-Look-Once version 5x (YOLOv5x) was conducted in two main phases. (A) Artificial intelligence (AI) Model Training Process: Glass slides were digitized using a smartphone, forming training, validation, and testing datasets. Malignant slides were annotated for abnormal cell clusters, whereas benign slides were treated as background. The model's performance on static images was first evaluated using the testing dataset (Evaluation 1). (B) Dataset Division: Flowchart illustrating case enrollment at Nippon Medical School Hospital from April 2017 to March 2023. Among 146 endometrial cytology cases, 96 (47 malignant, 49 benign) were allocated for training, validation, or testing datasets. Of the remaining 50 cases, 30 were used to define real-time detection thresholds at both the cell-cluster and slide levels, and the final 20 served as a test set to evaluate the AI model's performance against human evaluators and measure diagnostic time. (C) Real-Time Object Detection with Microscope. A charge-coupled device (CCD) camera was mounted on a microscope and linked to the trained model for real-time cluster detection. This phase included two evaluations: In Evaluation 2 (cell-cluster level), AI detections and confidence scores for specific pathologist-marked clusters were recorded to set thresholds for detecting abnormal cells. In Evaluation 3 (slide level), the total number of detected cell clusters exceeding these confidence thresholds was tallied, establishing the model's performance metrics under real-time conditions. Finally, in the last phase, the model's diagnoses for 20 new cases were compared with those of human evaluators (including pathologists) to assess AI's impact on diagnostic accuracy and diagnostic time.

The 146 cases were divided into: development set (96 cases) for You-Only-Look-Once version 5x (YOLOv5x; Ultralytics, https://github.com/ultralytics/yolov5) training and initial validation; real-time detection sets (30 cases) for optimizing confidence score (CS) and slide-level thresholds; and an independent test set (20 cases) for final evaluation (Fig. 1).

### Image acquisition and data annotation

To build a high-quality dataset for the AI model's training phase, we obtained high-resolution, manually focused images. Instead of using a WSI scanner, which can struggle with the thickness of cell clusters and is often unavailable in resource-limited settings, we used an iPhone SE (second generation, Apple Inc.) attached to an Olympus BX53 microscope via an adapter (i-NTER LENS; MICRONET Co.). This setup allowed us to acquire 4032 × 3024-pixel digital images at 20× objective magnification. The static images were used exclusively for training the AI model; the real-time inference system (described later) uses a CCD camera with a microscope.

The dataset comprised 3152 endometrial cytology images. On average, this corresponded to approximately 33.6 images per malignant case (*n* = 47, 1580 images total) and 32.1 images per benign case (*n* = 49, 1572 images total). Abnormal cell clusters in malignant cases were annotated as “malignant” in YOLO format using LabelImg (version 1.8.6), with final annotations confirmed by two board-certified pathologists. Examples of the resulting annotations applied to malignant clusters are provided in Supplementary Fig. S3. To prevent data leakage and overfitting, the 96 cases in the development set (47 malignant and 49 benign) were divided at the case level. The training set consisted of 78 cases (37 malignant, 41 benign; 2468 images), the validation set consisted of 9 cases (5 malignant, 4 benign; 308 images), and the test set consisted of 9 cases (5 malignant, 4 benign; 376 images), corresponding to an approximate 8:1:1 ratio. Data augmentation and random sampling were applied to the training set.

### AI model architecture and training

The YOLOv5x object detection model was chosen for its high image recognition performance and fast analysis capability, essential for real-time processing. Further details of the YOLOv5 architecture are illustrated in Supplementary Fig. S1. The model was pretrained on the 2017 Microsoft COCO dataset. The default data augmentation techniques provided by the YOLOv5 framework, including mosaic augmentation, random scaling/rotation, and color adjustments (Hue, Saturation, Value—HSV), blur and clade, were applied during training to enhance robustness.

Training was performed with a batch size of 4 and an image size of 640 × 640, using a stochastic gradient descent optimizer. We adopted the default hyperparameters from the standard YOLOv5 configuration (hyp.scratch-low.yaml) as a baseline for fine-tuning, as these are optimized for general object detection. The complete list of hyperparameters used, such as the initial learning rate (0.01), momentum (0.937), and weight decay (0.0005), is fully detailed in Supplementary Table S2.

The computational environment included an Intel Core i7 CPU and NVIDIA GeForce RTX3060 GPU (12GB), with Python 3.10.9 (Anaconda Distribution 2022.10), PyTorch 1.13.1, CUDA 11.6, and YOLOv5 (Ultralytics). Additional key libraries included scikit-learn 1.3.0 for statistical analysis and OpenCV for image processing. Input images (4032 × 3024 pixels) were automatically resized to 640 × 640 pixels by YOLOv5x with aspect ratio preservation and zero-padding (letterboxing).

### Evaluation 1: AI model performance evaluation with static images

The performance of the YOLOv5x model was evaluated using the hold-out test dataset. Precision, recall (sensitivity), F1-score, and mAP (mean average precision) were calculated at an IoU (intersection-over-union) threshold of 0.5. The precision-recall (PR) curves were used for performance evaluation. Evaluation metrics details are in Supplementary Methods S1.

### Real-time detection implementation (Evaluations 2 and 3)

**Fig. 2:**
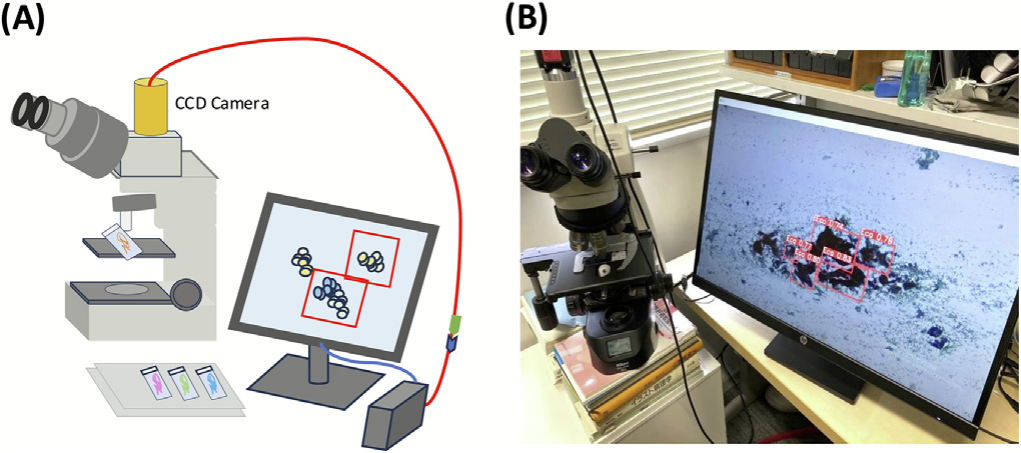
Real-time object detection setup. (A) Schematic illustration of the real-time detection system showing a standard microscope equipped with a CCD camera streaming images to a PC for real-time YOLOv5x inference. (B) Photograph of the live detection display illustrating the output of YOLOv5x inference with detected cell clusters marked by red bounding boxes and associated confidence scores. (For interpretation of the references to color in this figure legend, the reader is referred to the web version of this article.)

For real-time video detection, a CCD camera (JCS-HR5U, Canon Inc.) was attached to a Nikon ECLIPSE Ci microscope (LED illumination) via a 0.55× C-mount adapter. The microscope and CCD camera streamed live feed to a PC, displaying real-time bounding boxes and CS. The detection speed was monitored in frames per second (fps) (Fig. 2).

**Fig. 3:**
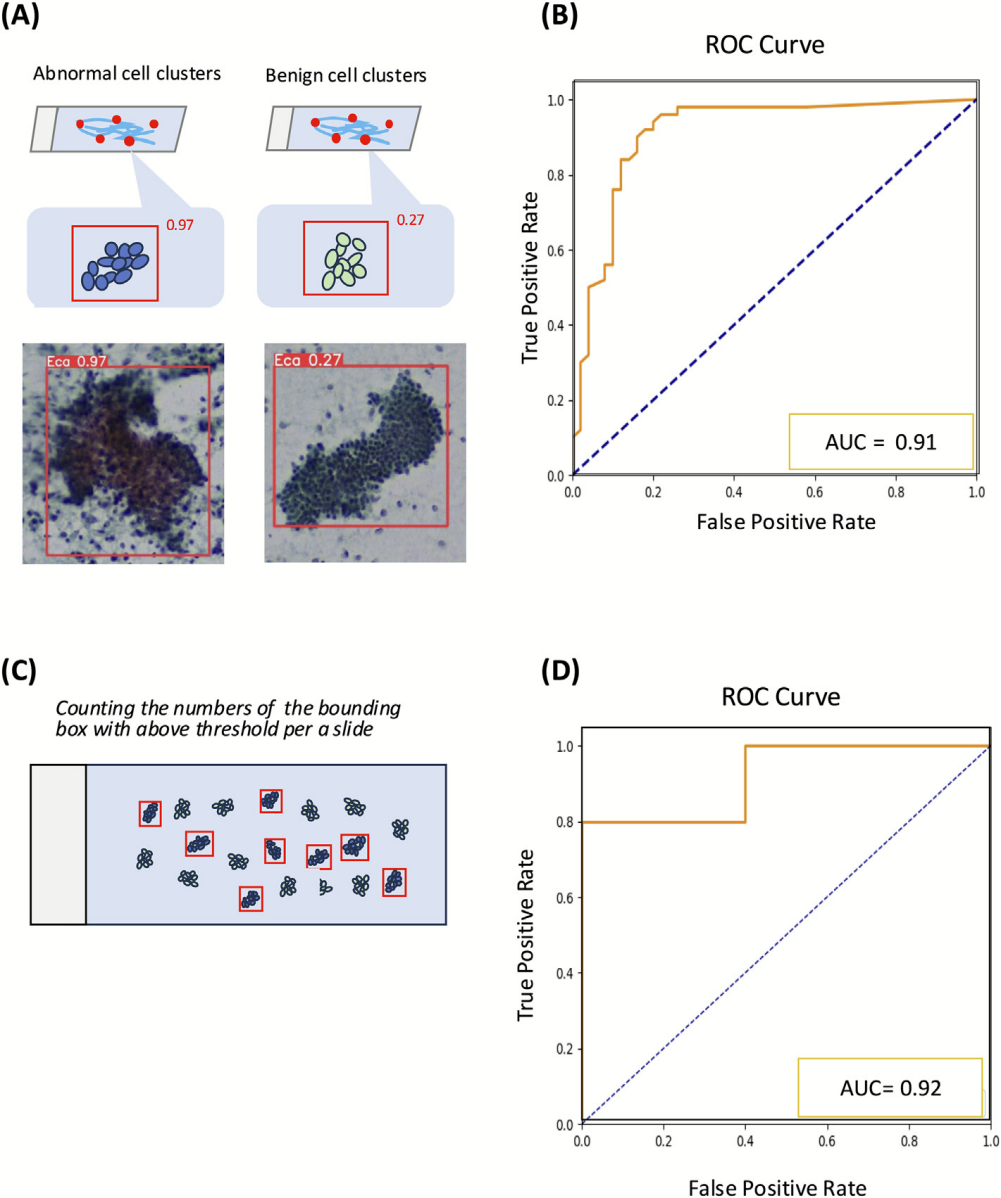
Determination of thresholds for real-time detection at cell-cluster and slide levels (Evaluations 2 and 3). (A) Cell-cluster-level evaluation. The upper panels illustrate examples of abnormal (left) and benign (right) cell clusters marked by a pathologist. The lower panels demonstrate corresponding YOLOv5x detection results with bounding boxes and associated confidence scores. (B) ROC curve (AUC = 0.91) generated from cell-cluster-level data to establish the optimal confidence score threshold for identifying abnormal clusters. (C) Slide-level evaluation schematic. The total number of cell clusters exceeding the confidence score threshold (determined in Evaluation 2) was counted for each slide. (D) ROC curve (AUC = 0.92) showing the determination of the slide-level diagnostic threshold based on the cluster counts.

For Evaluation 2 (cell-cluster level), we tested real-time detection on 20 slides (10 benign and 10 malignant). Two gynecological pathologists marked five distinct clusters in each slide with a marker pen. The model output bounding boxes for clusters with CSs ≥ 0.01. An ROC analysis yielded an AUC of 0.91, and we selected a final CS threshold of 0.225 to achieve optimal sensitivity and specificity (Fig. 3A and B).

For Evaluation 3 (slide level), a new set of 10 slides (5 benign, 5 malignant) was scanned for 4 min each in real-time. An ROC curve was generated using the total count of bounding boxes with CS ≥0.225 as the predictor, yielding an AUC of 0.92. From this curve, a cutoff of 220 bounding boxes per slide was selected as optimal, balancing sensitivity and specificity, for classifying a slide as “suspicious.” In this study, slides classified as “suspicious” by the AI model indicate a high likelihood of abnormal cells, warranting further clinical evaluation through biopsy or other diagnostic procedures (Fig. 3C and D).

### Diagnostic performance evaluation using an independent test set

Using the optimized CS threshold (≥0.225) and slide-level cutoff (≥220) determined in previous evaluations, the final diagnostic performance was evaluated on 20 independent cases (10 benign, 10 malignant) that were not included in any prior analyses. Each slide was scanned in real-time for 4 min, and the AI model classified it as “suspicious” based on the established thresholds. To assess inter-evaluator agreement, Cohen's kappa coefficient (κ) was calculated for the classifications made by each human evaluator and the AI model against the ground truth.

To assess the impact of AI assistance, each human evaluator subsequently re-examined the same test set (20 slides: 10 benign, 10 malignant) with AI support. To minimize learning or recall bias, the AI-assisted evaluation was conducted 2 weeks after the initial baseline (non-AI) evaluation, and the slide order was randomly shuffled. This washout period aimed to reduce the possibility that evaluators would recall specific cases. The diagnostic efficiency was measured by recording the time required for each evaluator to complete the evaluation of the test set.

## Results

### Evaluation 1: AI model performance on high-resolution static images

**Fig. 4:**
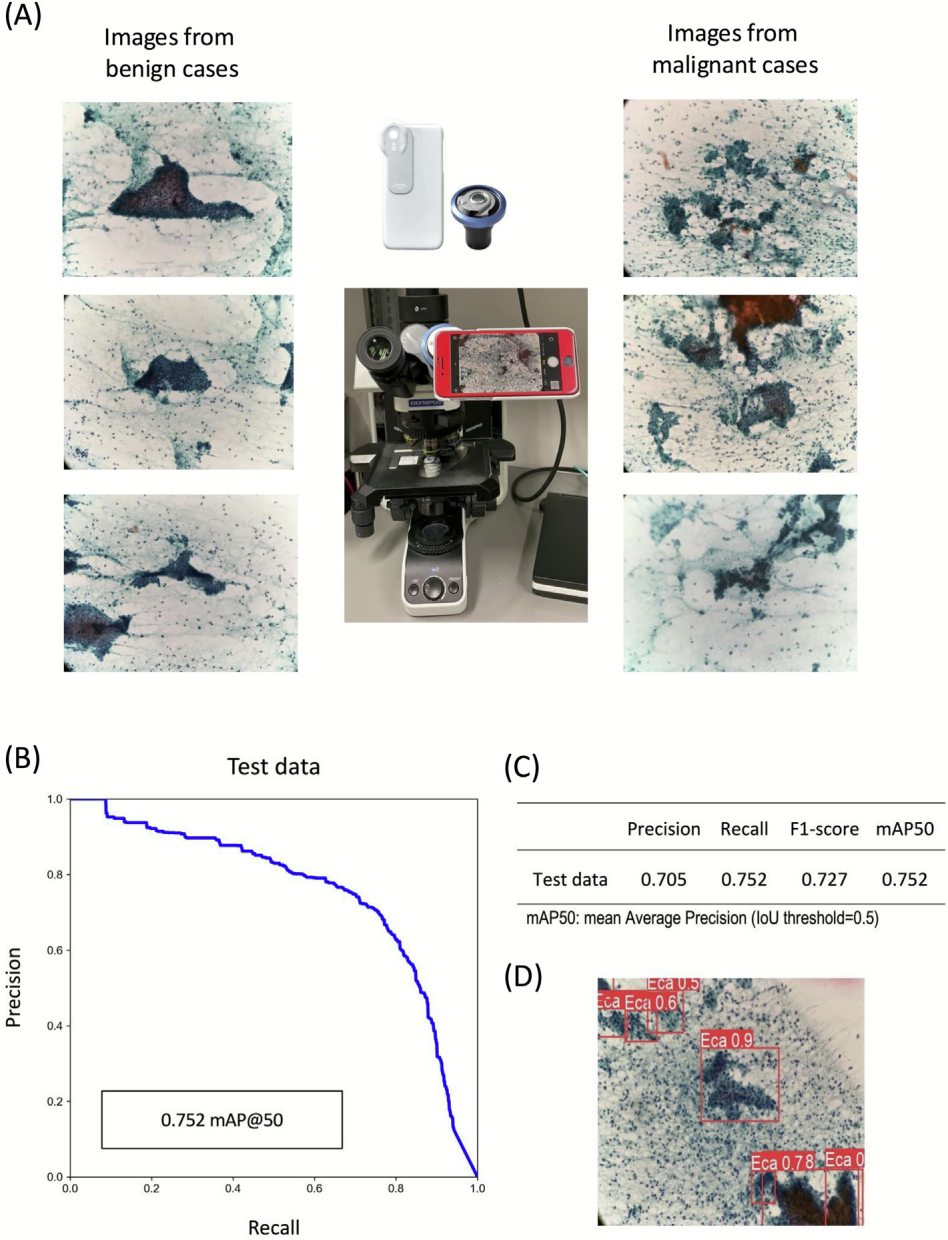
Performance evaluation of YOLOv5x on digitized endometrial cytology images. (A) Representative digitized images of benign (left column) and malignant (right column) cytology slides captured at 20× magnification using a smartphone camera mounted to a microscope via an adapter. (B) Precision–recall (PR) curve illustrating the YOLOv5x model performance at a 50% intersection-over-union (IoU) threshold, achieving a mean average precision (mAP@50) of 0.752 for the test dataset. (C) Summary of four key performance metrics (precision, recall, F1-score, and mAP@50) for the YOLOv5x model evaluated on the test dataset. (D) Example detection output from the test dataset, with abnormal cell clusters identified by the YOLOv5x model highlighted in red bounding boxes and labeled as endometrial carcinoma (Eca) with associated confidence scores. (For interpretation of the references to color in this figure legend, the reader is referred to the web version of this article.)

Initially, we evaluated the YOLOv5x model's performance using high-resolution static images obtained from a smartphone camera attached to a microscope. The representative cytology images (4032 × 3024 pixels) obtained at 20× magnification are shown in Fig. 4A. Despite significant cellular overlap typical in endometrial cytology preparations, these images provided sufficient resolution and clarity to train the YOLOv5x model directly without the need for patch extraction or additional preprocessing steps. The PR curve demonstrated a mean average precision (mAP@50) of 0.752 (Fig. 4B). Key evaluation metrics achieved were precision = 0.705, recall = 0.752, and F1-score = 0.727 (Fig. 4C). Although the performance metrics were modest, achieving values greater than 0.7 provided critical baseline validation. This baseline performance justified proceeding with experiments using a CCD camera-based real-time detection system, where object detection would need to function reliably under the more challenging conditions of live video streaming. The trained model accurately identified abnormal cell clusters and assigned them high CSs (Fig. 4D).

### Real-time detection performance

During real-time inference with the CCD camera attached to a microscope, the trained model achieved a detection speed of approximately 60 fps, exceeding the required 30 fps for smooth visualization.

### Diagnostic performance of AI and human evaluators

Using the optimized thresholds (CS ≥0.225 and ≥220 bounding boxes per slide), the AI model was evaluated on 20 independent cases (10 benign, 10 malignant). Under these conditions, the model achieved an accuracy of 0.85, precision of 0.82, recall of 0.90, and F1-score of 0.86 (Table 1).

**Table 1:** Performance metrics and diagnostic times with and without AI assistance.

		Pathol-1	Pathol-2	Pathol-3	Stud-1	Stud-2	Stud-3	Stud-4	YOLOv5x
Accuracy									0.85
AI-assist	(−)	0.80	0.70	0.65	0.80	0.80	0.65	0.75	
	(+)	0.75	**0.75**	**0.80**	**0.85**	**0.85**	0.65	0.60	
Precision									0.82
AI-assist	(−)	0.80	0.67	0.64	0.80	0.80	0.64	0.73	
	(+)	0.69	0.67	**0.75**	**0.82**	**0.82**	0.64	0.60	
Recall									0.90
AI-assist	(−)	0.80	0.80	0.70	0.80	0.80	0.70	0.80	
	(+)	**0.90**	**1.00**	**0.90**	**0.90**	**0.90**	0.70	0.60	
F1 score									0.86
AI-assist	(−)	0.80	0.73	0.67	0.80	0.80	0.67	0.76	
	(+)	0.78	**0.80**	**0.82**	**0.86**	**0.86**	0.67	0.60	
Diagnostic time (s)									4800
AI-assist	(−)	3014	2544	6600	4458	4681	4593	2940	
	(+)	**1800**	**2040**	**2700**	**1860**	**3354**	**2764**	**2460**	

Note: AI-assist (−), diagnosis without AI assistance; AI-assist (+), diagnosis with AI assistance; Pathol-1, gynecological pathologist; Pathol-2 and Pathol-3, non-gynecological pathologists; Stud-1 and Stud-2, medical students with AI and cytology training; Stud-3 and Stud-4, medical students with minimal cytology training and AI exposure. **Bold** indicates improved performance metrics or reduced diagnostic time with AI assistance (+) compared to without AI assistance (−) for human evaluators. Due to the limited sample size (7 evaluators, 20 cases), results are descriptive without statistical testing. The YOLOv5x row shows AI-only performance without human evaluators.

We compared these results with those of three pathologists and four medical students, each performing binary classification (“suspicious” or “not suspicious”) on the same test set. Among the human evaluators, Pathol-1 (a gynecological pathology specialist) achieved accuracy, precision, recall, and F1-score of 0.80. Agreement with the ground truth (histological diagnosis) was assessed using Cohen's kappa coefficient (κ), an indicator of agreement beyond chance. The AI model showed the highest concordance (κ = 0.70), followed by Pathol-1 and two senior students (Stud-1, Stud-2) at κ = 0.60. A heatmap of these kappa values is shown in Supplementary Fig. S2.

### Impact of AI-assisted implementation on diagnostic performance

**Fig. 5:**
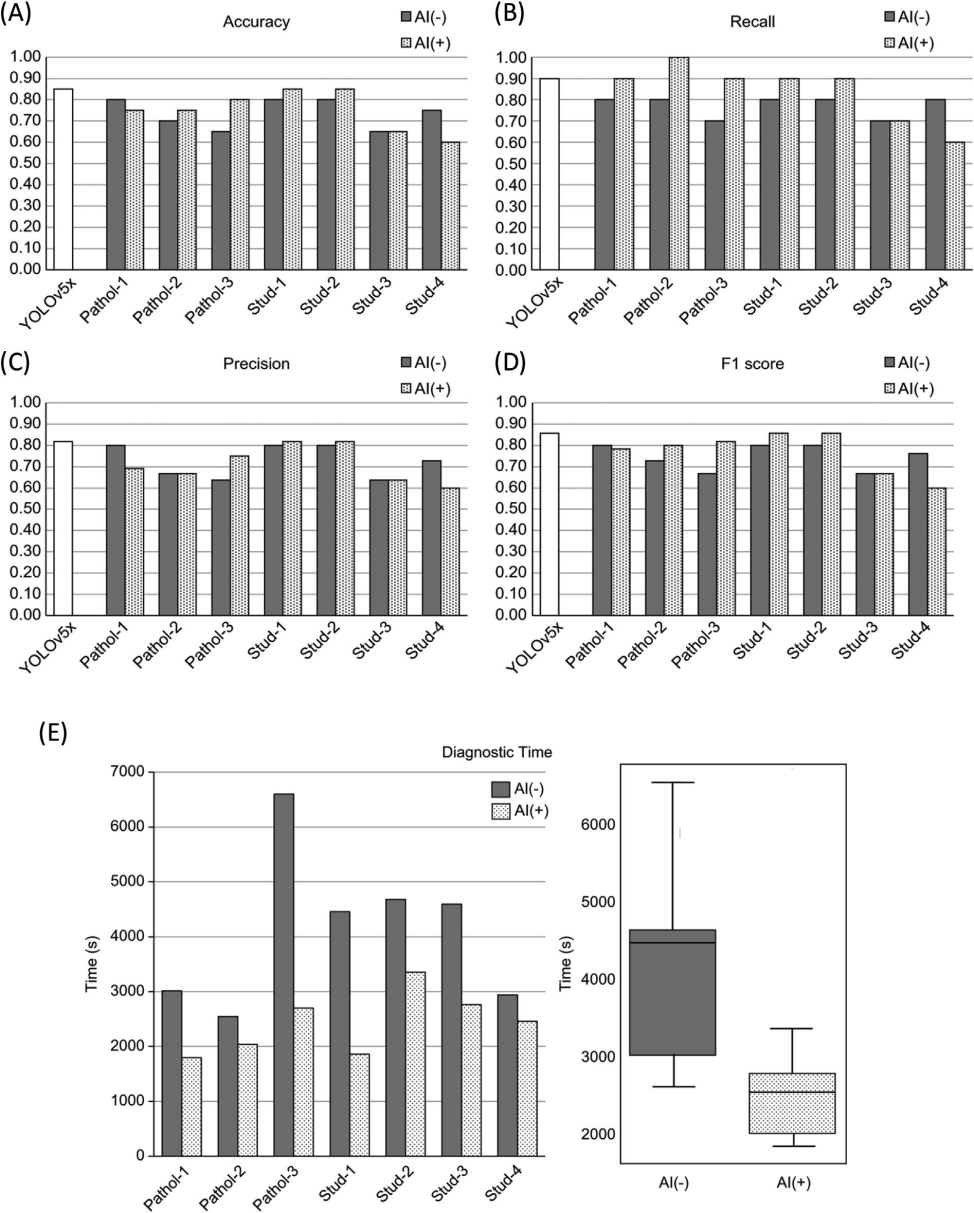
Impact of AI assistance on diagnostic performance and efficiency of human evaluators. (A–D) Comparison of diagnostic performance metrics—(A) Accuracy, (B) Recall, (C) Precision, and (D) F1-score—between evaluations conducted without AI assistance [AI(−)] and with AI assistance [AI(+)]. Evaluators: Pathol-1 (gynecological pathologist with minimal AI experience); Pathol-2 and Pathol-3 (non-gynecological pathologists with minimal AI experience); Stud-1 and Stud-2 (medical students trained in AI modeling and cytology); Stud-3 and Stud-4 (medical students with minimal AI and cytology exposure). (E) Diagnostic time (in seconds) for each evaluator without AI [AI(−)] and with AI [AI(+)] assistance (bar graph). Box plot summarizes the distribution of diagnostic times across all evaluators. Given the limited sample size (7 evaluators, 20 cases), these data represent descriptive summaries, and statistical significance tests were not performed.

Each human evaluator re-examined the test set with AI support. To minimize learning or recall bias, the AI-assisted phase was conducted 2 weeks after the baseline (non-AI) phase, with slides randomly shuffled. As shown in Fig. 5 and Table 1, AI assistance affected each evaluator differently. Non-gynecological pathologists (Pathol-2 and Pathol-3) showed accuracy improvements (0.70 → 0.75 and 0.65 → 0.80, respectively). Medical students experienced in AI and cytology (Stud-1 and Stud-2) also improved (0.80 → 0.85). However, the gynecological pathology specialist (Pathol-1) showed a slight decrease (0.80 → 0.75). Students with minimal training and AI experience (Stud-3 and Stud-4) exhibited minimal or negative changes in certain metrics (Fig. 5C, D). Given the small sample size, these findings are purely descriptive and not statistically validated.

### Time measurement for diagnosis

We measured the time required for each of the seven evaluators to complete their assessments with and without AI. As shown in Fig. 5E and Table 1, the median time without AI was 4458 s, which decreased to 2460 s with AI assistance—a 44.8% reduction. The interquartile range also narrowed (from 1741 s without AI to 904 s with AI). All evaluators showed a similar pattern of time reduction. Due to the small sample size, these findings are descriptive, and formal statistical testing was not performed.

## Discussion

This proof-of concept study demonstrates the feasibility and clinical potential of a real-time AI-assisted system for endometrial cytology using widely available equipment. By integrating the YOLOv5x model with a standard microscope and an affordable CCD camera, promising results were observed in diagnostic efficiency—reducing diagnostic time by nearly half—and diagnostic accuracy, particularly among non-specialist evaluators, although these findings require confirmation in larger studies due to the limited sample size. These findings suggest that real-time AI support could effectively address challenges related to the shortage of expert diagnosticians, improving diagnostic workflow efficiency and diagnostic quality, especially in resource-limited settings.^15^^,^^16^

Whereas endometrial cytology is not the global standard for screening, it is a technique widely employed in Japan, where its high diagnostic performance has been extensively documented,^6^^,^^7^^,^^9^ and for these reasons, remains a highly valued screening tool in Japan, serving as an excellent proof-of-concept for developing affordable, real-time AI assistance for complex cytology. However, diagnostic interpretation can be challenging because of thick, overlapping, and morphologically heterogeneous cell clusters. Training expert diagnosticians is resource-intensive, and widespread adoption of digital pathology via WSI remains difficult due to high cost and complex infrastructure requirements.^10^^,^^11^ Additionally, traditional WSI-based AI methods typically rely on extensive z-stack imaging and substantial data storage, making them impractical for resource-limited clinical facilities.^12^

A key feature of our system is its immediate visual feedback during live microscopic examination. As our results indicated, the impact of this feedback varied by expertise, which represents a key finding of this study. The observed improvement in diagnostic accuracy among non-specialists suggests that our system could have potential impacts on clinical workflows, including reduced inter-institutional diagnostic variability and accelerated training processes.^15^^,^^16^ The modest impact on the specialist's accuracy, however, could be interpreted in the context of a potential “ceiling effect.” Our dataset was challenging, with a high proportion of well-differentiated carcinomas exhibiting subtle cytological features. The specialist's high baseline accuracy of 80% on these cases may have limited the potential for further AI-driven improvement. This finding reinforces that the primary utility of our system lies not in surpassing expert-level performance, but rather in two other key areas: (1) the substantial 44.8% reduction in diagnostic time across *all* evaluators and (2) helping less-experienced users approach expert-level accuracy. We believe this demonstrates the system's significant potential to improve workflow efficiency and standardize care, which was our primary objective.

Compared to Google's Augmented Reality Microscope (ARM)—another AI system operating without WSI—our method has practical advantages.^17^ ARM, despite being more affordable than traditional WSI scanners, still requires specialized hardware investments, limiting widespread adoption. In contrast, our system employs existing microscopy equipment, widely available and affordable CCD cameras, and standard-specification PCs, thus potentially lowering implementation barriers in clinical settings. Furthermore, because our focus is primarily on cytological screening, characterized by cellular overlap complexities, we believe that initial AI implementation is most suitable in the screening domain, rather than in definitive diagnosis for tissue specimens.

Successful AI implementation in clinical environments depends not only on technological performance but also on clinician acceptance and workflow integration.^18^ Our system directly addresses key acceptance factors: it maintains clinician autonomy by providing visual guidance instead of automated diagnoses, integrates smoothly with familiar microscopy procedures, and offers immediate feedback to enhance rather than replace expert judgment. The observed variation in AI assistance efficacy across user expertise is notable. For instance, the lack of improvement for junior students (Stud-3 and Stud-4) with minimal exposure suggests that the AI tool is most effective when augmenting foundational cytological knowledge, rather than replacing it. This highlights the importance of tailored user training and suggests future developments of adaptive systems that dynamically respond to various skill levels.

The operator-dependent nature, which requires manual stage movement and focus, is a deliberate design choice to ensure low cost and accessibility. Unlike fully automated WSI-based systems, which require expensive scanners and complex digital infrastructure, our system is intended as a real-time visual assistance tool for resource-limited settings where WSI is not feasible.

The proposed workflow, therefore, is not a fully automated screening tool but an integrated assistant for the standard microscopy process. It is optimized for the typical 10× objective magnification used in routine screening. The model's role is to provide immediate visual guidance on ambiguous cell clusters, thereby aiming to reduce diagnostic uncertainty and time, particularly for non-specialists or trainees. This hybrid, human-in-the-loop approach, which leverages the strengths of both the human expert and the AI, is central to our system's value, addressing the challenges of accessibility and workflow integration.

Regarding model selection, our choice of the YOLO framework was deliberate. We acknowledge that YOLO models are well-established in medical imaging, as confirmed by recent comprehensive reviews.^13^ However, these same reviews highlight that significant challenges remain, particularly in detecting small or occluded objects and the high computational demands required for training and inference.

Our study's unique contribution is the practical application of this established technology to a domain—endometrial cytology—that inherently features these exact challenges (i.e., complex 3D, overlapping cell clusters), while simultaneously addressing the accessibility barrier. Unlike studies predicated on WSI, our system was intentionally designed to operate using standard, affordable lab hardware.

Within this framework, we specifically chose YOLOv5x over more recent versions (e.g., YOLOv8 or v12) based on practical and clinical considerations. Whereas newer variants may offer state-of-the-art accuracy, they often require substantially more computational resources, complicating real-time inference on standard lab hardware used in our low-cost design. In contrast, YOLOv5x is well-established in clinical applications and provides a proven balance between high accuracy, stable real-time inference performance, and compatibility with affordable computing environments.^13^ Given our primary goal—developing a clinically feasible, cost-effective AI support tool—YOLOv5x remains an optimal choice for this proof-of-concept implementation. Nevertheless, future comparative studies evaluating newer YOLO variants with expanded data and computational resources may further enhance performance in diagnostic accuracy and speed.

Practical considerations for real-world deployment include periodic software updates, continuous user training, computational resource maintenance, and rigorous quality-control protocols. To ensure sustainable clinical performance, these operational aspects must be integrated into the clinical workflow. Additionally, future research should explore explainable AI methods—such as heatmaps, attention mechanisms, and feature attribution—to enhance clinician trust and comprehension of AI outputs, aligning closely with the cognitive processes employed by pathologists.

## Limitations

This study has several important limitations, primarily concerning data and generalizability. First, the sample size was small and data collection was limited to a single institution. This was particularly true for the independent test set (*n* = 20), which was intentionally kept small to mitigate the significant risk of evaluator fatigue, given the complex and time-consuming nature of endometrial cytology (requiring 1–2 h for the 20 cases, as shown in Fig. 5). This decision, while ensuring data fidelity, restricts the statistical power of the human–AI comparison.

Furthermore, all benign cases in this study were leiomyomas, a selection bias resulting from our study design requiring post-operative hysterectomy specimens as the gold-standard. Additionally, the model was trained on institution-specific protocols and on static images acquired manually by pathologists, introducing a potential subjectivity bias (e.g., capturing suspicious fields). Consequently, these factors limit the generalizability of our findings to diverse datasets, staining techniques, and other benign endometrial conditions (e.g., polyps, inflammation).

A second category of limitations relates to the model's scope and performance. The current model functions only as visual assistance for identifying abnormal cell clusters without differentiating malignant from benign lesions (a definitive classification). Moreover, the model's performance on static images (mAP@50 = 0.752) was modest. This was partly attributable to our annotation strategy of labeling only malignant clusters. However, our primary objective for the static evaluation was not to maximize performance, but to establish a sufficient baseline to justify proceeding with our main goal: evaluating the real-time video detection workflow (Evaluations 2 and 3), which was the true test of the system's practical utility.

Future research must address these limitations, primarily by validating the model's generalizability with larger, multi-institutional datasets and standardized image acquisition protocols. Concurrently, enhancing the underlying static model's performance and clinical utility is a clear priority. This will involve moving beyond binary classification to incorporate robust, detailed, multiclass annotations (e.g., “inadequate cellularity,” “normal,” “polyp,” “‘inflammation”), a path this proof-of-concept study has now established.

Beyond these direct improvements, the real-time, low-cost framework itself holds potential for broader applications. Adapting this AI framework to other cytological domains with similar diagnostic challenges—such as cellular overlapping and 3D structures where rapid assessment is critical, including conventional PAP smears or Fine Needle Aspiration Cytology specimens—represents an important avenue for enhancing the value and utility of this approach.

## Conclusions

This study demonstrates the technical feasibility of real-time AI-based detection and visualization of abnormal cell clusters in endometrial cytology using standard lab equipment. Rather than delivering definitive diagnostic decisions, our system provides immediate visual guidance to pathologists during routine microscopic examinations. By leveraging affordable and widely accessible hardware, this approach could substantially reduce barriers to AI integration into cytology workflows, especially in resource-limited settings. However, further multicenter validation studies and prospective clinical evaluations are necessary to confirm the clinical impact of this real-time AI approach. Our findings thus lay a foundation for developing practical, user-friendly AI tools that support expert cytological assessment without replacing human judgment.

## CRediT authorship contribution statement

M.T. conceived and designed the study, collected samples, developed methodology, and wrote the article. S. Tanaka and I.S. developed methodology and wrote the article. E.T. and Y.T. performed data analysis and reviewed the article. S. Takakuma, Y.K., and S.K. provided technical support. Y.T. and A.S. reviewed the study design and article. All authors read and approved the final manuscript.

## Consent for publication

Not applicable.

## Ethics approval and consent to participate

This study received ethical approval from the Institutional Review Board of Nippon Medical School (approval number: M-2022-041) and adhered to relevant laws, institutional guidelines, and the Declaration of Helsinki. Given the retrospective nature of the study, informed consent was waived.

## Declaration of generative AI and AI-assisted technologies in the writing process

During the preparation of this work, the authors used Gemini (Google) in order to improve readability, clarity, and grammatical accuracy. After using this tool/service, the authors reviewed and edited the content as needed and take full responsibility for the content of the published article.

## Funding

This work was supported by the 10.13039/501100001691Japan Society for the Promotion of Science (JSPS) [Grant-in-Aid for Scientific Research (C), No. 23K08900]; and the 10.13039/501100001700Ministry of Education, Culture, Sports, Science and Technology (MEXT) through the Initiative for Realizing Diversity in the Research Environment.

## Declaration of competing interest

The authors declare that they have no known competing financial interests or personal relationships that could have appeared to influence the work reported in this article.

## Data Availability

The data that support the findings of this study are not publicly available due to privacy concerns and ethical considerations. Access to the data can be requested by qualified researchers and institutions for purposes of replication, verification, and further research. Interested parties may request access to the data by contacting the corresponding author, Mika Terasaki, at mterasaki@nms.ac.jp, or the institution at Nippon Medical School Hospital, Sendagi 1-1-5, Bunkyo-ku, Tokyo, Japan. Requests for data access will be subject to review and approval to ensure compliance with all relevant ethical and legal standards regarding patient data privacy and confidentiality. The YOLOv5x code and hyperparameters that support the findings of this study are available from GitHub (https://github.com/ahp-aig/yolo_for_endometrial_cytology).
