# Supplementary material for: An integrated system from microscopy to AI for real-time object detection in endometrial cytology
Source: J Pathol Inform. 2025 Dec 31;20:100541. doi: 10.1016/j.jpi.2025.100541 (PMC12859783; doi:10.1016/j.jpi.2025.100541)
Supplement: Supplementary material — Supplementary Information [file mmc1.pdf]

Supplementary Information

Supplementary Tables

Supplementary Table S1. Distribution and Median Ages of Malignant and Benign Diagnoses

		AI model training	Real-time object detection under a microscope	
		Training, validation, and testing (*n* = 96)	Cell-cluster and slide-level assessment (*n* = 30)	Verification of diagnostic concordance with and without AI (*n* = 20)
**Malignant**	Median age (range)	57 (31–82)	58 (38–83)	54 (28–77)
	Number of cases	47	15	10
Endometrioid carcinoma	Grade 1	24	10	5
	Grade 2	17	3	4
	Grade 3	5	0	0
Serous carcinoma		1	2	1
**Benign**	Median age (range)	47 (37–73)	46 (30–57)	45 (38–51)
	Number of cases	49	15	10
Leiomyoma		49	15	10

Supplementary Table S2. Key hyperparameters and training options used for YOLOv5x

**Parameter**	**Value**	**Description**
weights	yolov5x.pt	Pretrained model checkpoint for fine-tuning
epochs	200	Maximum number of epochs
batch_size	4	Number of images per training batch
imgsz	640	Input image size (letterbox to 640×640)
optimizer	SGD	Optimizer (Stochastic Gradient Descent)
lr0	0.01	Initial learning rate
lrf	0.01	Final learning rate factor (cosine schedule if used)
momentum	0.937	Momentum for SGD
weight_decay	0.0005	L2 regularization factor
patience	100	Early-stopping patience (training is halted if there is no improvement in validation for 100 consecutive epochs)
hsv_h	0.015	Max hue shift for color augmentation
hsv_s	0.7	Max saturation shift
hsv_v	0.4	Max value (brightness) shift
degrees	0.0	Random rotation in degrees
translate	0.1	Random translation up to 10%
scale	0.5	Random scale ±50%
flipud	0.0	Vertical flip probability = 0%
fliplr	0.5	Horizontal flip probability = 50%
mosaic	1.0	Probability of mosaic augmentation (set to 1.0, i.e., always on)
mixup	0.0	Mixup augmentation disabled

blur	0.01	Custom blur augmentation
median_blur	0.01	Custom median-blur augmentation
gray	0.01	Convert to grayscale probability
clahe	0.01	Contrast Limited Adaptive Histogram Equalization
random_gamma	0.0	Probability of gamma correction
brightness_contrast	0.0	Probability of brightness-contrast augmentation
image_compression	0.0	Probability of compression artifact

Notes: Training was performed on a GeForce RTX 3060 GPU with 12 GB VRAM.
The model automatically stopped at epoch 143 due to early stopping (patience=100).
All data augmentations and hyperparameters were configured based on the YOLOv5 opt.yaml file.

Supplementary Methods

Supplementary Methods S1. Metrics for evaluating the performance of YOLOv5x trained on static images of endometrial cytology

- Precision quantifies the proportion of **correct** (or **true**) positive identifications among all **predicted** positive identifications made by the model. It is expressed as follows:

$$\text{Presicion} = \frac{TP}{TP + FP}$$

where TP and FP denote true positives and false positives, respectively.

- Recall, also known as sensitivity, is proportion of **correct** positive identifications among all **actual** positive instances to all actual positive instances. It is expressed as

$$\text{Recall} = \frac{TP}{TP + FN}$$

where FN denotes false negatives.

- F1 score is the harmonic mean of precision and recall, and is defined as follows:

$$\text{F1 Score} = \frac{2 \times Precision \times Recall}{Precision + Recall}$$

- Precision–recall (PR) curves plot precision against recall. The area under the PR curve is often referred to as the average precision (AP), which is a standard metric in object detection by averaging the maximum precision values across various recall thresholds. AP is defined as

$$\text{AP} = \sum_{k=1}^{m} p(k) \vartriangle r(k)$$

where k denotes the rank in the sorted list of detections (e.g., sorted by descending confidence score), $p(k)$ denotes the precision at the k -th detection cutoff k, and $\Delta r(k)$ denotes the change in recall from items $k - 1$ to k. m is the total number of detections in the list.

- mAP is the average of AP, that is,

$$mAP = \frac{1}{n}\sum_{i=1}^{n} AP(i)$$

where n denotes the number of classes, and $AP(i)$ denotes the AP value for class i. Because only one class of objects was trained, the AP and mAP in this study were equivalent.

Supplementary Figures

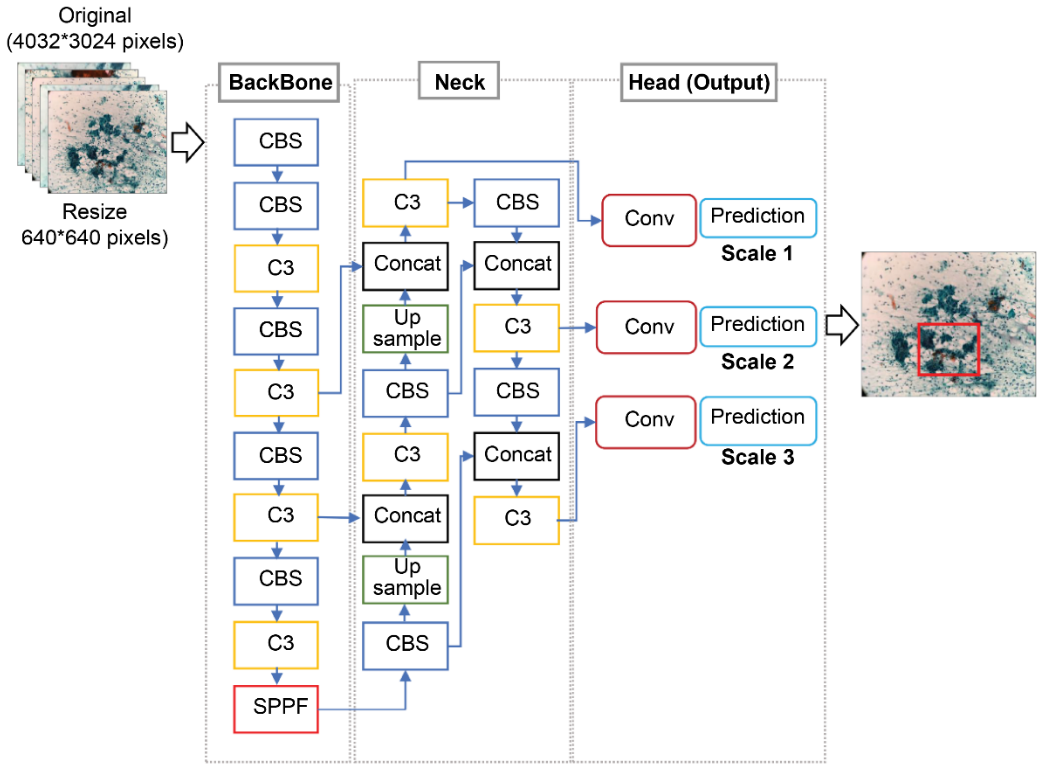

Supplementary Figure S1. Architecture of YOLOv5, detailing the process from image input to detection. The original images, at a resolution of 4,032 × 3,024 pixels, were first resized to 640 × 640 pixels during preprocessing. The YOLOv5 architecture comprises three main components: a backbone, a neck, and a head. The backbone employs a cross-stage partial network (CSP) with DarkNet53 to enhance feature-map processing efficiency. It achieves this by splitting the input into two parallel paths, thus preserving computational resources and maintaining information diversity. The neck component uses a path aggregation network (PANet) to propagate features more effectively and integrates spatial pyramid pooling fusion to handle variable input sizes. Finally, the head produces predictions at multiple scales, ensuring robust detection across different object scales and contexts.

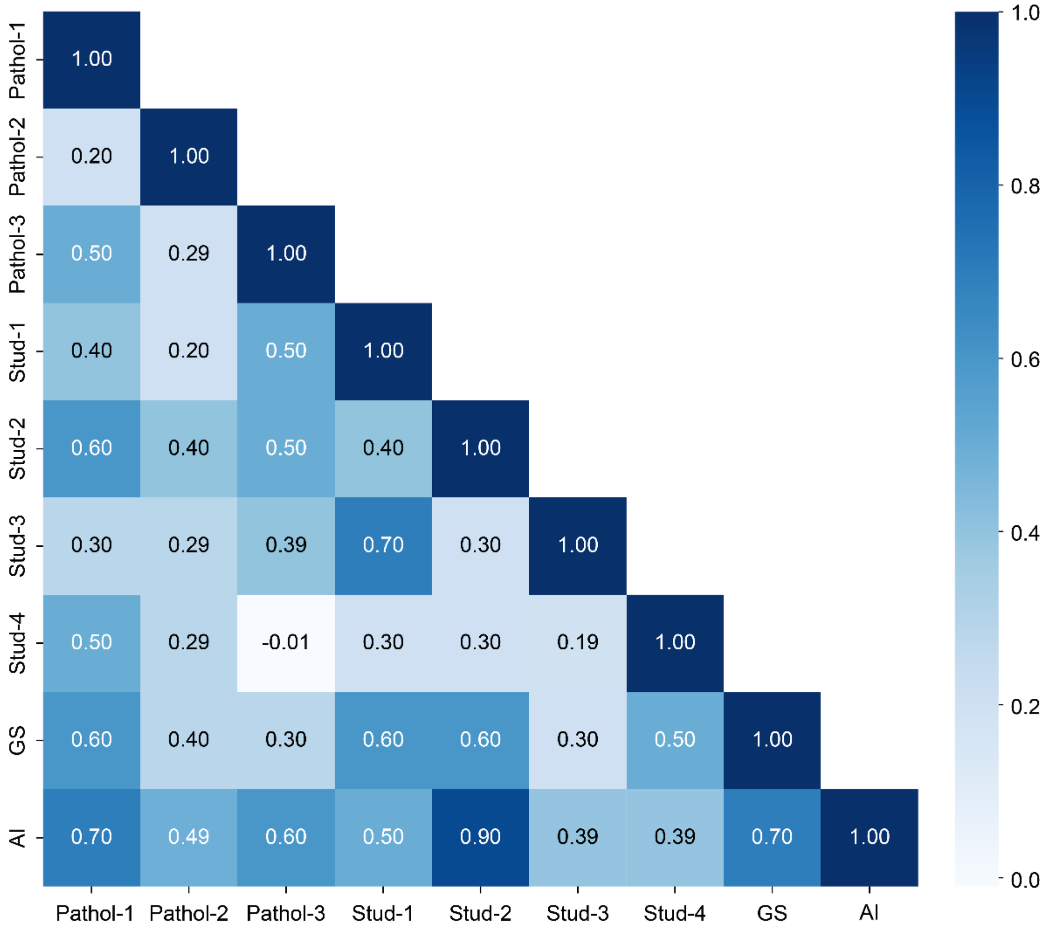

Supplementary Figure S2. Heatmap of Cohen's kappa coefficients comparing diagnostic concordance among evaluators in preoperative cytopathology. The color scale ranges from –0.01 (no agreement) to 1.00 (perfect agreement). **Abbreviations:** GS, Gold standard; Pathol-1, a gynecologic pathologist; Pathol-2 and Pathol-3, two nongynecologic pathologists; Stud-1 and Stud-2, two senior medical students; Stud-3 and Stud-4, two junior students; AI, YOLOv5x model.

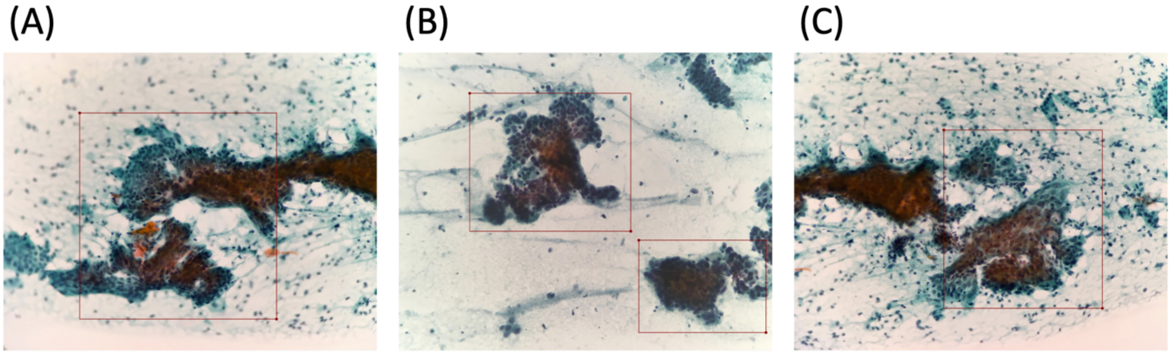

Supplementary Figure S3. Examples of annotated malignant clusters used for model training. These images represent typical bounding box annotations applied to malignant cell clusters from various endometrial cancer cases used in this study. Identification of these clusters as malignant was based on the consensus of two pathologists specializing in gynecologic pathology. Red bounding boxes indicate the annotated regions. Benign clusters and background elements were not annotated. Specific histological subtypes were not differentiated during the annotation process for this model.
